# Long-Horizon Constraint-Aware Collaborative Scheduling for Multiple Phased-Array Radars Using Mamba Temporal Encoding and Structured Hybrid Actions

**DOI:** 10.3390/s26154772

**Published:** 2026-07-27

**Authors:** Jianan Liu, Jie Xu, Wenge Xing, Mingrui Li

**Affiliations:** 1 Nanjing Research Institute of Electronics Technology, Nanjing 210039, China; muyuliu1105@163.com (J.L.); whesly5287@163.com (W.X.); 2 School of Information and Communication Engineering, Dalian University of Technology, Dalian 116024, China; 2905450254@mile.dlut.edu.cn

**Keywords:** Mamba, phased-array radar network, collaborative scheduling, constraint-aware reinforcement learning, hybrid action space, radar resource management, temporal sequence encoding

## Abstract

Phased-array radar networks require real-time collaborative scheduling of search, tracking, and identification tasks under coupled resource, beam, time-window, and power constraints. Current decisions affect future residual power, task refresh intervals, and tracking uncertainty, making long-horizon scheduling particularly challenging. This paper proposes CS-Mamba, a long-horizon constraint-aware collaborative scheduling framework for homogeneous multiple phased-array radars. The scheduling problem is formulated as a finite-horizon constrained decision process with structured hybrid actions, where the discrete component represents radar–task matching and the continuous component represents transmit-power allocation. A Mamba-based temporal encoder is introduced to summarize long scheduling histories with linear sequence complexity. Based on the encoded representation, the scheduler predicts task priorities, constructs a masked radar–task bipartite graph, solves a constrained maximum-weight matching problem, and projects raw transmit powers onto the feasible power domain. In addition, an action-dependent radar model is incorporated to link transmit power, effective SNR, detection probability, measurement noise, and tracking covariance. The model is trained using behavioral cloning from constraint-aware heuristic trajectories followed by actor–critic fine-tuning. Experiments on the proposed MRSched-Bench show that CS-Mamba improves the normalized cost-effectiveness score from 0.62 to 0.78 compared with MAPPO in the Medium scenario, while reducing end-to-end decision latency from 24.5 ms to 12.8 ms per step. Additional ablation studies verify the contributions of temporal encoding, structured matching, feasible power projection, and two-stage training.

## 1. Introduction

Phased-array radar systems play a critical role in modern surveillance and target monitoring by enabling rapid electronic beam steering and flexible multi-task execution. In practical deployments, multiple radars must collaboratively allocate sensing resources among search, tracking, and identification tasks while satisfying operational requirements such as power budgets, beam availability, task deadlines, periodic coverage, and cooperative tracking consistency. As radar networks increase in scale and mission complexity, collaborative scheduling becomes a key challenge for improving resource utilization and mission effectiveness.

Existing scheduling approaches can be broadly divided into optimization-based methods and learning-based methods. Traditional optimization methods, such as mixed-integer programming, heuristic search, genetic algorithms, and tabu search, can generate high-quality schedules in small-scale or static scenarios. However, their computational cost increases rapidly under dynamic multi-radar coordination, nonlinear detection and tracking models, and long-horizon resource coupling. Reinforcement learning methods have recently shown promising adaptability for sequential resource management. Nevertheless, they still face three major difficulties in radar scheduling. First, long scheduling horizons introduce temporal coupling among residual power, tracking covariance, task refresh intervals, and electromagnetic interference. Second, radar scheduling naturally involves hybrid decisions, including discrete radar–task matching and continuous transmit-power allocation. Third, many learning-based methods handle constraints mainly through reward penalties, which may lead to infeasible exploration and reduced training efficiency.

To address these issues, this paper proposes CS-Mamba, a long-horizon constraint-aware collaborative scheduling framework for multiple homogeneous phased-array radars. Instantaneous hard constraints are embedded into action generation through graph-edge masking, constrained matching, and feasible-domain power projection, while mission-level requirements such as cooperative tracking and periodic refresh are optimized as soft penalties. The method combines Mamba temporal encoding, structured hybrid action generation, two-stage policy optimization, and an action-dependent radar performance model.

This work focuses on homogeneous multiple phased-array radar networks. All radar nodes share the same sensing model and waveform configuration, while their residual power, beam accessibility, local interference, and task visibility may vary over time. Heterogeneous radar networks with different frequency bands, waveform libraries, and sensing modalities are beyond the scope of this study and will be investigated in future work.

The main contributions are summarized as follows:

(1) We formulate collaborative scheduling of multiple homogeneous phased-array radars as a finite-horizon constraint-aware decision problem with structured hybrid actions. The formulation explicitly distinguishes instantaneous hard constraints, long-term resource constraints, soft mission-level requirements, and terminal performance metrics.
(2) We develop a radar-specific structured hybrid action-generation mechanism. The neural scheduler first predicts task priorities and radar–task compatibility scores; these scores are then converted into executable radar–task assignments by graph-edge masking and constrained maximum-weight matching.
(3) We introduce a feasible transmit-power projection layer and an action-dependent radar performance model, which links transmit power to effective signal-to-noise ratio, detection probability, measurement noise, and tracking covariance. This establishes a physical causal path from continuous power actions to scheduling utility.
(4) We combine Mamba temporal encoding with two-stage policy optimization. Behavioral cloning provides a stable initialization from constraint-aware heuristic trajectories, while actor–critic fine-tuning improves beyond the heuristic expert under the same feasible action-generation pipeline.
(5) We construct a reproducible MRSched-Bench simulation protocol and report not only normalized cost-effectiveness score and latency but also hard-constraint violation rate, soft-constraint satisfaction rate, task completion rate, power utilization, and tracking error.

## 2. Related Work

### 2.1. Radar Resource Management and Cooperative Radar Scheduling

Radar resource management has been extensively studied in phased-array radar networks, distributed MIMO radars, and cognitive radar systems [1,2,3]. Classical methods typically formulate task scheduling, node selection, dwell-time allocation, waveform selection, or power allocation as optimization problems. Mixed-integer programming, dynamic programming, heuristic search, genetic algorithms, simulated annealing, and tabu search have been used to generate feasible schedules under beam, time-window, and resource constraints. These methods are interpretable and effective in small-scale scenarios, but their scalability is limited when nonlinear detection models, recursive tracking covariance updates, dynamic interference, and long-term resource coupling are considered simultaneously [4,5,6,7,8].

Cooperative radar tracking and resource allocation methods further investigate node selection and power allocation to improve detection probability and tracking accuracy. However, many of these studies focus on short-horizon allocation or single-step optimization, rather than long-horizon collaborative scheduling with coupled discrete-continuous actions. In contrast, this paper considers radar–task matching, transmit-power allocation, task refresh status, residual power evolution, and tracking uncertainty within a unified sequential decision framework.

### 2.2. Constrained and Hybrid-Action Reinforcement Learning

Constrained Markov decision processes and constrained policy optimization provide principled formulations for sequential decision-making under constraints. However, practical engineering constraints are often handled through Lagrangian relaxation, penalty terms, or post hoc correction, which may still allow infeasible exploration during training. In radar scheduling, invalid actions, such as assigning an inaccessible task to a radar or allocating power beyond the remaining budget, are physically meaningless and should be avoided before execution [7,8].

Hybrid-action reinforcement learning, including parameterized-action methods such as P-DQN, addresses problems involving both discrete action choices and continuous action parameters. Nevertheless, the discrete component of radar scheduling is not a simple categorical action, but a structured radar–task matching matrix with capacity, beam, time-window, and exclusiveness constraints. Directly learning such a high-dimensional hybrid action space is difficult. The proposed CS-Mamba framework, therefore, uses a hierarchical action-generation mechanism that separates task prioritization, constrained radar–task matching, and feasible power allocation [9,10,11,12].

### 2.3. Sequence Models for Decision Making

Sequence models have recently been introduced into reinforcement learning and offline decision modeling. Decision Transformer formulates reinforcement learning as conditional sequence modeling using states, actions, and return-to-go. State-space models, especially Mamba, provide an efficient alternative to attention-based Transformers by using selective state updates with linear sequence complexity. Such models are suitable for long-horizon problems where historical information must be summarized efficiently.

Although each radar scheduling slot is short, the decision horizon contains hundreds of slots. Residual power, tracking covariance, task refresh intervals, and interference statistics evolve cumulatively across slots. Therefore, a policy based only on the current state or a short memory may satisfy instantaneous feasibility but still cause future resource exhaustion or refresh violations. LSTM and GRU are efficient but may struggle to preserve slow-varying resource and constraint information over long horizons. Transformer encoders can model long-range dependency but introduce quadratic complexity with sequence length. Mamba provides a selective state-space mechanism with linear sequence complexity and data-dependent state updates, which is suitable for long-horizon radar scheduling under real-time latency requirements [13,14,15,16,17].

### 2.4. Positioning of This Work

The proposed method is not intended to claim novelty in Mamba architectures, constrained MDPs, maximum-weight matching, or behavioral cloning individually, which is shown in Table 1. Instead, its contribution lies in integrating these components into a radar-specific scheduling pipeline with explicit physical modeling and executable constraint handling. Compared with constrained policy optimization methods, CS-Mamba does not rely solely on Lagrangian penalties to satisfy instantaneous engineering constraints. Compared with parameterized-action reinforcement learning methods such as P-DQN, the discrete action is not a simple categorical index but a structured radar–task matching matrix. Compared with Decision Transformer or Decision Mamba, CS-Mamba is not purely an offline sequence model; it combines temporal encoding with online actor–critic fine-tuning and feasibility-enforcing execution layers. Compared with classical radar resource-allocation methods, the proposed framework simultaneously considers long-horizon task scheduling, radar–task matching, transmit-power allocation, and tracking-performance feedback [18,19,20,21].

## 3. Method

### 3.1. Overall Framework

**Figure 1:**
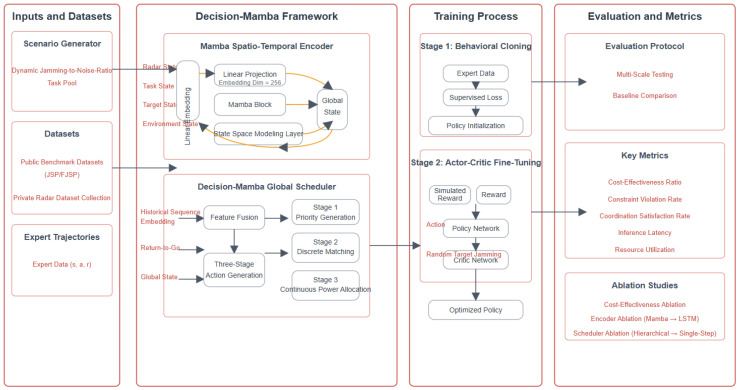
CS-Mamba for Multi-Radar Collaborative Scheduling.

The overall framework of the proposed CS-Mamba scheduler is shown in Figure 1. The framework consists of four key components: constraint-aware scheduling formulation, Mamba-based temporal encoding, hierarchical hybrid action generation, and two-stage policy optimization [22,23,24,24]. At each scheduling slot, radar resources, task states, target information, and electromagnetic conditions are first organized as a temporal state sequence. The Mamba encoder then compresses long-horizon scheduling history into a compact representation. Based on this representation, the scheduler sequentially predicts task priorities, performs feasible radar–task matching, and allocates continuous transmit power. Hard constraints are embedded into the action generation process, while soft mission requirements are optimized through reward penalties.

Different from conventional reinforcement learning schedulers that usually adopt purely discrete or continuous actions, the proposed framework explicitly models the structured hybrid nature of phased-array radar scheduling [25,26,27,28,29,30]. The discrete matching matrix determines which radar executes which task, while the continuous power matrix determines how much transmit power is assigned to each selected radar–task pair. This design follows the engineering logic of “matching first and allocating later”, making the generated schedules more executable under practical radar constraints.

### 3.2. Problem Formulation

#### 3.2.1. Radar Network and Task Model

We consider a homogeneous phased-array radar network consisting of $N_{R}$ radar nodes and $N_{T}$ candidate tasks or targets. The scheduling horizon contains *K* decision slots, and the duration of each slot is Δt. At slot *k*, the scheduler observes the global state $s_{k}$ and generates a structured hybrid action $a_{k}$. The decision process is modeled as a finite-horizon constrained Markov decision process:

(1) M=〈S,A,P,r,γ〉,k=1,…,K,

where S is the state space, A is the structured action space, $P(s_{k+1}|s_{k},a_{k})$ is the transition kernel, $r(s_{k},a_{k})$ is the slot-level reward, and γ is the discount factor. The objective is

(2) $$\max_{\theta }E_{\tau ∼\pi_{\theta }}\left[\sum_{k=1}^{K}\gamma^{k-1}r\left(s_{k},a_{k}\right)\right],\;s.t.\;a_{k}\in A_{hard}\left(s_{k}\right).$$

The action consists of a discrete radar–task matching matrix and a continuous transmit-power matrix:

(3) $$a_{k}=\left(X_{k},P_{k}\right),$$

where

(4) $$X_{k}=\left\{x_{r,t,k}\right\}\in {\left\{0,1\right\}}^{N_{R}\times N_{T}},\;P_{k}=\left\{p_{r,t,k}\right\}\in R_{+}^{N_{R}\times N_{T}}.$$

Here, $x_{r,t,k}=1$ indicates that radar *r* is assigned to task *t* at slot *k*, and $p_{r,t,k}$ denotes the corresponding transmit power.

#### 3.2.2. State Variables and Transition Dynamics

The state vector contains radar resource status, task status, electromagnetic environment information, and historical scheduling statistics:

(5) $$s_{k}=\left[S_{k}^{res},S_{k}^{task},S_{k}^{env},S_{k}^{hist}\right].$$

All continuous variables are normalized before being fed into the neural scheduler, which is shown in Table 2. Residual power is divided by the initial power budget, target position is scaled by the surveillance-region size, target velocity is scaled by the maximum target speed, tracking covariance is log-normalized, and jamming and clutter levels are normalized in the dB domain.

The residual power evolves according to

(6) $$P_{r,k+1}^{rem}=P_{r,k}^{rem}-\sum_{t=1}^{N_{T}}p_{r,t,k}\Delta t.$$

The feasible projection layer ensures $P_{r,k+1}^{rem}\geq 0$ for all radars and slots.

The task refresh interval is updated as

(7) $$\delta_{t,k+1}=\left\{\begin{array}{cc} 0, & \mathrm{if}\;\sum_{r=1}^{N_{R}}x_{r,t,k}>0,\\ \delta_{t,k}+1, & \mathrm{otherwise}. \end{array}\right.$$

The target state follows a constant-velocity motion model:

(8) $$\xi_{t,k+1}=F\xi_{t,k}+w_{t,k},\;w_{t,k}∼N\left(0,Q_{t}\right),$$

where $\xi_{t,k}$ denotes the kinematic state of target *t*, *F* is the state-transition matrix, and $Q_{t}$ is the process-noise covariance. The predicted tracking covariance is

(9) $$P_{t,k|k-1}=FP_{t,k-1|k-1}F^{⊤}+Q_{t}.$$

#### 3.2.3. Constraint Classification

We distinguish hard feasibility constraints from soft mission-level requirements. Hard constraints must be satisfied at every scheduling slot and define the feasible action set. Long-term resource constraints couple current actions with future feasible sets through residual power and task-refresh states. Soft mission-level requirements are allowed to be violated but incur reward penalties. Terminal constraints are used only for episode-level evaluation.

The instantaneous hard constraints include radar exclusiveness, task capacity, residual power, beam accessibility, task time-window feasibility, and power-matching consistency:

(10) $$\sum_{t=1}^{N_{T}}x_{r,t,k}\leq 1,\;\forall r,k,$$

(11) $$\sum_{r=1}^{N_{R}}x_{r,t,k}\leq b_{t,k},\;\forall t,k,$$

(12) $$\sum_{t=1}^{N_{T}}p_{r,t,k}\leq P_{r,k}^{rem},\;\forall r,k,$$

(13) $$x_{r,t,k}\leq g_{r,t,k},\;\forall r,t,k,$$

(14) $$x_{r,t,k}=0,\;\forall r,t,\;\forall k\notin K_{t},$$

(15) $$0\leq p_{r,t,k}\leq P_{r}^{max}x_{r,t,k},\;\forall r,t,k.$$

Soft mission-level requirements include the minimum cooperative radar requirement and maximum task refresh interval:

(16) $$\phi_{t,k}^{tr}=\beta_{tr}\max\left(0,M_{t}-n_{t,k}\right),\;n_{t,k}=\sum_{r=1}^{N_{R}}x_{r,t,k},$$

(17) $$\phi_{t,k}^{per}=\beta_{per}\max\left(0,\delta_{t,k}-\Pi_{t}\right).$$

Therefore, the proposed method guarantees feasibility with respect to instantaneous hard constraints and residual-power constraints, while cooperative tracking and periodic-refresh requirements are treated as soft mission-level objectives. This distinction is also reflected in the evaluation metrics.

### 3.3. Reward Function and Evaluation Metric

The slot-level training reward is defined as a difference-type objective:

(18) $$r_{k}=U_{k}-\lambda_{c}C_{k}-\lambda_{\phi }\Phi_{k},$$

where $U_{k}$ is the task utility, $C_{k}$ is the resource cost, and $\Phi_{k}$ is the soft-constraint penalty.

The task utility is

(19) $$U_{k}=\sum_{t=1}^{N_{T}}I_{t,k}\left[w_{t}^{d}P_{d,t,k}+w_{t}^{tr}\exp\left(-\alpha_{t}\epsilon_{t,k}\right)\right],$$

where $I_{t,k}=1\left[\sum_{r=1}^{N_{R}}x_{r,t,k}>0\right]$ indicates whether task *t* is executed, $P_{d,t,k}$ is the fused detection probability, and $\epsilon_{t,k}$ is the tracking error.

The resource cost is

(20) $$C_{k}=\sum_{r=1}^{N_{R}}\sum_{t=1}^{N_{T}}x_{r,t,k}\left(\lambda_{p}p_{r,t,k}+\lambda_{\tau }\tau_{r,t}\right),$$

where $\tau_{r,t}$ denotes the dwell or execution cost associated with radar–task pair (r,t).

The soft-constraint penalty is

(21) $$\Phi_{k}=\sum_{t=1}^{N_{T}}\left(\phi_{t,k}^{tr}+\phi_{t,k}^{per}\right).$$

The training reward and the reported evaluation metric are different. The reward is used for slot-level policy optimization, while the reported normalized cost-effectiveness score is an episode-level normalized metric. For an episode with horizon *K*, we define

(22) $$U_{ep}=\sum_{k=1}^{K}U_{k},\;C_{ep}=\sum_{k=1}^{K}C_{k},\;\Phi_{ep}=\sum_{k=1}^{K}\Phi_{k}.$$

The normalized terms are

(23) $${\bar{U}}_{ep}=\frac{U_{ep}}{U_{ep}^{max}+\epsilon },\;{\bar{C}}_{ep}=\frac{C_{ep}}{C_{ep}^{max}+\epsilon },\;{\bar{\Phi }}_{ep}=\frac{\Phi_{ep}}{\Phi_{ep}^{max}+\epsilon }.$$

The normalized cost-effectiveness score is defined as

(24) $$CE=\mathrm{clip}\left({\bar{U}}_{ep}-\lambda_{C}{\bar{C}}_{ep}-\lambda_{\Phi }{\bar{\Phi }}_{ep},0,1\right).$$

The final reported value is averaged over all test scenarios and random seeds:

(25) $$CE_{avg}=\frac{1}{N_{seed}N_{test}}\sum_{i=1}^{N_{seed}}\sum_{j=1}^{N_{test}}CE_{i,j}.$$

Unless otherwise stated, all normalized cost-effectiveness scores reported in the experiments are averaged over five random seeds and all test scenarios.

### 3.4. Mamba-Based Temporal Encoder

Although each scheduling slot is short, the decision horizon may contain hundreds of slots. Residual power, tracking covariance, task refresh intervals, and interference statistics evolve cumulatively across slots. Therefore, a policy based only on the current state or a short memory may satisfy instantaneous feasibility but still cause future resource exhaustion or refresh violations.

At slot *k*, the historical state sequence is denoted as

(26) $$S_{1:k}=\left(s_{1},s_{2},\ldots ,s_{k}\right)\in R^{k\times D_{s}}.$$

Each state vector is projected into an embedding space:

(27) $$x_{k}=W_{e}s_{k}+b_{e},\;x_{k}\in R^{D_{emb}}.$$

The embedded sequence is processed by stacked Mamba blocks. A simplified selective state-space update is written as

(28) $$A_{k}=\mathrm{SelectA}\left(x_{k}\right),\;B_{k}=\mathrm{SelectB}\left(x_{k}\right),\;C_{k}=\mathrm{SelectC}\left(x_{k}\right),$$

(29) $$h_{k}=A_{k}h_{k-1}+B_{k}x_{k},\;z_{k}=C_{k}h_{k}.$$

The encoded temporal representation is

(30) $$z_{k}=E_{\mathrm{Mamba}}\left(S_{1:k}\right).$$

LSTM and GRU are efficient but may struggle to preserve slow-varying resource and constraint information over long horizons, which is shown in Table 3. Transformer encoders can model long-range dependency but introduce quadratic complexity with sequence length. Mamba provides a selective state-space mechanism with linear sequence complexity and data-dependent state updates, making it suitable for long-horizon radar scheduling under real-time latency requirements [31,32,33,34,35,36,37].

### 3.5. Decision-Mamba Scheduler

The neural scheduler does not directly sample the final radar–task matching matrix. Instead, it outputs pre-decision variables, including task-priority scores, radar–task compatibility scores, and raw transmit powers. The final executable action is obtained by deterministic feasibility-enforcing layers.

The pre-decision variables are denoted as

(31) $$u_{\theta ,k}=\left(\pi_{\theta ,k},W_{\theta ,k},P_{\theta ,k}^{raw}\right),$$

where $\pi_{\theta ,k}$ is the task-priority distribution, $W_{\theta ,k}=\left\{w_{r,t,k}\right\}$ is the radar–task compatibility weight matrix, and $P_{\theta ,k}^{raw}=\left\{p_{r,t,k}^{raw}\right\}$ is the raw power matrix.

The executable matching matrix is generated by

(32) $$X_{k}=M\left(W_{\theta ,k},s_{k}\right),$$

where M denotes the constrained maximum-weight matching operator.

The executable power matrix is obtained by feasible-domain projection:

(33) $$P_{k}=P\left(P_{\theta ,k}^{raw},X_{k},s_{k}\right).$$

Therefore, the final action is

(34) $$a_{k}=\Gamma_{\theta }\left(s_{k}\right)=\left(X_{k},P_{k}\right).$$

The constrained matching and power projection modules are treated as feasibility-enforcing execution layers rather than differentiable stochastic policy layers. Gradients are not propagated through the combinatorial matching solver. During behavioral cloning, supervision is applied to the pre-decision variables, while during actor–critic fine-tuning the environment receives only the feasibility-projected executable action.

Stage 1: Task Priority Prediction.

The model first predicts a priority distribution over tasks. The task-priority distribution is predicted as

(35) $$\pi_{\theta ,k}=\mathrm{softmax}\left(\frac{\mathrm{FC}(f_{k})}{\tau }\right),$$

where $f_{k}$ is the fused decision feature and τ is the temperature coefficient.

The radar–task compatibility coefficient is defined as

(36) $$\eta_{r,t,k}=\lambda_{tr}\frac{\epsilon^{ref}}{\epsilon_{r,t,k}+\epsilon^{ref}}+\lambda_{snr}\frac{\gamma_{r,t,k}^{eff}}{\gamma_{r,t,k}^{eff}+\gamma^{ref}}.$$

The unmasked radar–task weight is

(37) $$w_{r,t,k}=\pi_{\theta ,k}\left(t\right)\eta_{r,t,k}.$$

Stage 2: Constraint-Aware Radar–Task Matching.

Action masking is implemented as edge masking in the radar–task bipartite graph. The feasibility mask is defined as

(38) $$m_{r,t,k}=1\left[g_{r,t,k}=1\right]1\left[k\in K_{t}\right]1\left[P_{r,k}^{rem}>0\right].$$

The masked matching weight is

(39) $${\tilde{w}}_{r,t,k}=\left\{\begin{array}{cc} w_{r,t,k}, & m_{r,t,k}=1,\\ -M, & m_{r,t,k}=0, \end{array}\right.$$

where *M* is a sufficiently large positive constant used in implementation.

The matching matrix is obtained by solving

(40) $$X_{k}=\arg\max_{X}\sum_{r=1}^{N_{R}}\sum_{t=1}^{N_{T}}{\tilde{w}}_{r,t,k}x_{r,t,k},$$

subject to

(41) $$X\in X_{hard}\left(s_{k}\right).$$

Here, $X_{hard}\left(s_{k}\right)$ denotes the set of matching matrices satisfying radar exclusiveness, task capacity, beam feasibility, and task time-window constraints.

Stage 3: Feasible Power Allocation.

For each matched radar–task pair, the neural scheduler first predicts a raw transmit power:

(42) $$p_{r,t,k}^{raw}=P_{r}^{max}\cdot \mathrm{sigmoid}\left({\mathrm{FC}}_{p}\left(f_{k},r,t\right)\right).$$

The executable power matrix is obtained by Euclidean projection:

(43) $$P_{k}=\arg\min_{P}\frac{1}{2}\sum_{r=1}^{N_{R}}\sum_{t=1}^{N_{T}}{\left(p_{r,t,k}-p_{r,t,k}^{raw}\right)}^{2},$$

subject to

(44) $$0\leq p_{r,t,k}\leq P_{r}^{max}x_{r,t,k},\;\forall r,t,k,$$

(45) $$\sum_{t=1}^{N_{T}}p_{r,t,k}\leq P_{r,k}^{rem},\;\forall r,k.$$

For the single-beam setting, where each radar is assigned to at most one task at each slot, the projection has the following closed-form solution:

(46) $$p_{r,t,k}=x_{r,t,k}\min\left\{\max\left(p_{r,t,k}^{raw},0\right),P_{r}^{max},P_{r,k}^{rem}\right\}.$$

If multi-beam scheduling is enabled, the projection becomes a box-constrained simplex projection for each radar and can be solved by a sorting-based water-filling algorithm with complexity

(47) $$O(|T_{r}\left(k\right)|\log|T_{r}\left(k\right)|),$$

where $T_{r}\left(k\right)=\left\{t|x_{r,t,k}=1\right\}$. In the current implementation, the projection layer is used as a feasibility-enforcing execution operator.

Two-Stage Policy Optimization.

The policy is trained using a two-stage strategy. The first stage performs behavioral cloning from expert trajectories generated by a constraint-aware rolling heuristic. The second stage performs actor–critic fine-tuning in the simulation environment.

For an expert trajectory, the return-to-go is computed as

(48) $$G_{k}^{E}=\sum_{k^{\prime }=k}^{K}\gamma^{k^{\prime }-k}r\left(s_{k^{\prime }}^{E},a_{k^{\prime }}^{E}\right).$$

During behavioral cloning, the model is supervised on task-priority labels and raw power targets:

(49) $$L_{BC}=E_{\left(s_{k},a_{k}^{E},G_{k}^{E}\right)∼D_{E}}\left[L_{prio}+\lambda_{pow}L_{power}\right].$$

The task-priority loss is

(50) $$L_{prio}=-\sum_{t=1}^{N_{T}}y_{t,k}^{E}\log\pi_{\theta ,k}\left(t\right),$$

where $y_{t,k}^{E}$ is the expert task-priority label. The power loss is

(51) $$L_{power}=\sum_{r=1}^{N_{R}}\sum_{t=1}^{N_{T}}x_{r,t,k}^{E}{\left(p_{r,t,k}^{raw}-p_{r,t,k}^{E}\right)}^{2}.$$

During online fine-tuning, the future return is unavailable. To avoid an abrupt distribution shift from expert return-to-go to critic value, we use a warm-up return-conditioning strategy:

(52) $$G_{k}^{online}=\rho_{n}{\hat{G}}_{k}^{BC}+\left(1-\rho_{n}\right)\mathrm{stopgrad}\left(V_{\phi }\left(s_{k}\right)\right),$$

where

(53) $$\rho_{n}=\max\left(0,1-n/N_{warm}\right).$$

The critic value is detached before being used as an actor input, so actor gradients do not update the critic through the conditioning pathway.

The critic is trained by minimizing

(54) $$L_{critic}=E_{\tau ∼\pi_{\theta }}\left[{\left(V_{\phi }\left(s_{k}\right)-{\hat{R}}_{k}\right)}^{2}\right],$$

where ${\hat{R}}_{k}$ is the bootstrapped return. The actor is updated using advantage-based policy optimization on the pre-decision variables, while the environment receives only the feasibility-projected action.

## 4. Experiments

### 4.1. Experimental Setup

Experiments were conducted on a workstation equipped with an Intel Xeon Gold 6330 CPU, an NVIDIA RTX 4090 GPU with 24 GB memory, and 64 GB RAM. The implementation was based on Python 3.10, PyTorch 2.1, CUDA 11.8, and a Gymnasium-based multi-radar scheduling simulator. Unless otherwise stated, all results are reported as mean ± standard deviation over five random seeds.

The proposed method is evaluated on MRSched-Bench, a simulation benchmark for homogeneous multiple phased-array radar scheduling, which is shown in Table 4. The benchmark includes Small, Medium, and Large scenarios with different radar numbers, task loads, scheduling horizons, and interference intensities [38,39,40,41,42].

The reward and evaluation coefficients are summarized in Table 5. These values are kept fixed for all methods.

### 4.2. Baselines and Implementation Details

We compare CS-Mamba with classical heuristic methods, optimization-based methods, reinforcement-learning methods, hybrid-action reinforcement-learning methods, and sequence-modeling decision methods. The baselines include EDF, RM, GA, Tabu Search, MILP/ILP, DQN, PPO, MAPPO, QMIX, MADDPG, P-DQN, Decision Transformer, and Decision Mamba.

EDF denotes Earliest Deadline First, which schedules tasks according to the closest deadline. RM denotes Rate Monotonic scheduling, which assigns higher priorities to tasks with shorter refresh periods.

All learning-based baselines use the same state variables, reward function, train/test splits, and random seeds when applicable. Constraint masks are provided to the baselines whenever their action formulation supports masking.

The MAPPO implementation follows the centralized-training decentralized-execution paradigm, which is shown in Table 6. Each radar is treated as an agent. The centralized critic observes the global radar–task state, while each decentralized actor observes the corresponding radar state and a global task summary. The actor network consists of two hidden layers with dimensions 256 and 256, followed by separate heads for task selection and power prediction. The critic network consists of three hidden layers with dimensions 512, 256, and 128 [43,44,45,46,47].

The MILP/ILP baseline is implemented as a rolling-window approximate optimization method rather than an exact solver for the full nonlinear finite-horizon problem. The original scheduling problem contains nonlinear detection fusion, SNR-dependent measurement noise, recursive tracking covariance updates, and long-term resource coupling, which cannot be directly encoded as a tractable MILP without approximation. Therefore, the MILP/ILP baseline uses linearized utility terms, a rolling scheduling window, and a fixed per-slot time budget. Gurobi is used with a time limit of 100 ms per slot in the Small scenario. The reported MILP/ILP result should be interpreted as a tractable optimization baseline rather than the global optimum of the original nonlinear scheduling problem [48,49].

### 4.3. Constraint and Task-Level Metrics

The hard-constraint violation rate is defined as

(55) $$HVR=\frac{\sum_{k=1}^{K}1\left[a_{k}\notin A_{hard}\left(s_{k}\right)\right]}{K}.$$

The soft-constraint satisfaction rate is defined as

(56) $$SSR=1-\frac{\sum_{k=1}^{K}\sum_{t=1}^{N_{T}}1\left[n_{t,k}<M_{t}\;\mathrm{or}\;\delta_{t,k}>\Pi_{t}\right]}{KN_{T}}.$$

The task completion rate is

(57) $$TCR=\frac{\sum_{t=1}^{N_{T}}1\left[\mathrm{task}\;t\;\mathrm{completed}\right]}{N_{T}}.$$

The power utilization rate is

(58) $$PUR=\frac{\sum_{k=1}^{K}\sum_{r=1}^{N_{R}}\sum_{t=1}^{N_{T}}p_{r,t,k}}{\sum_{r=1}^{N_{R}}P_{r}^{bud}}.$$

Constraint and task-level performance in the Medium scenario is shown in Table 7.

### 4.4. Results on MRSched-Bench

The proposed method is first evaluated on the dedicated MRSched-Bench benchmark, which contains Small, Medium, and Large scenarios. These scenarios share the same state definition, action semantics, reward structure, and constraint mechanism, while differing in radar number, task scale, scheduling horizon, task load, and interference intensity. The Small scenario is used for comparison with optimization-based methods, the Medium scenario serves as the main benchmark, and the Large scenario evaluates scalability under high-load and constraint-intensive conditions [50,51].

As shown in Table 8, the proposed method achieves the best normalized cost-effectiveness score across all scenario scales. In the Medium scenario, which serves as the main benchmark, our method improves the normalized cost-effectiveness score from 0.62 obtained by MAPPO to 0.78, corresponding to a relative improvement of 25.8%. Compared with Decision Transformer and Decision Mamba, the proposed method also achieves higher scheduling quality, indicating the effectiveness of combining Mamba temporal encoding with constraint-aware hybrid action generation.

In terms of real-time performance, the proposed method achieves the lowest end-to-end latency among learning-based methods. In the Medium scenario, the decision latency is reduced from 24.5 ms for MAPPO and 28.4 ms for Decision Transformer to 12.8 ms. In the Large scenario, the latency advantage becomes more evident, demonstrating the scalability of the linear-complexity Mamba encoder under long-horizon scheduling.

**Figure 2:**
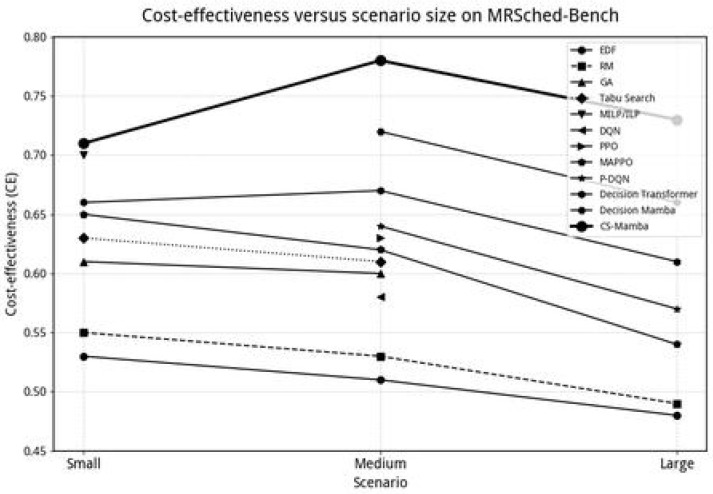
Comparison of normalized cost-effectiveness scores under different scenario scales.

**Figure 3:**
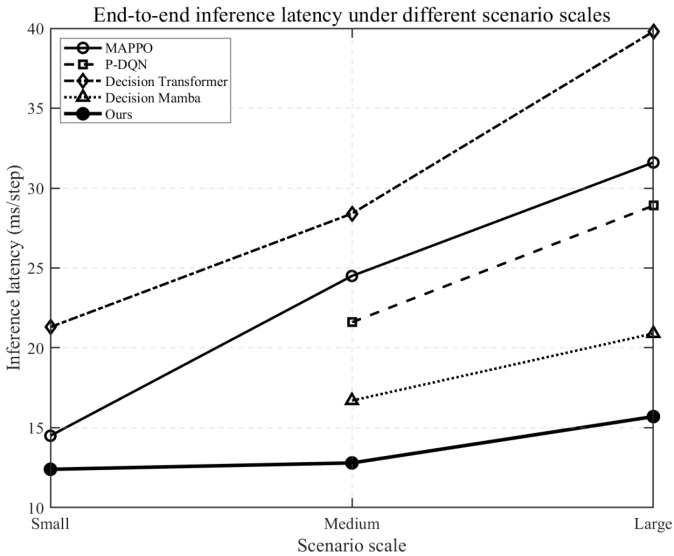
Comparison of end-to-end decision latency under different scenario scales.

**Figure 4:**
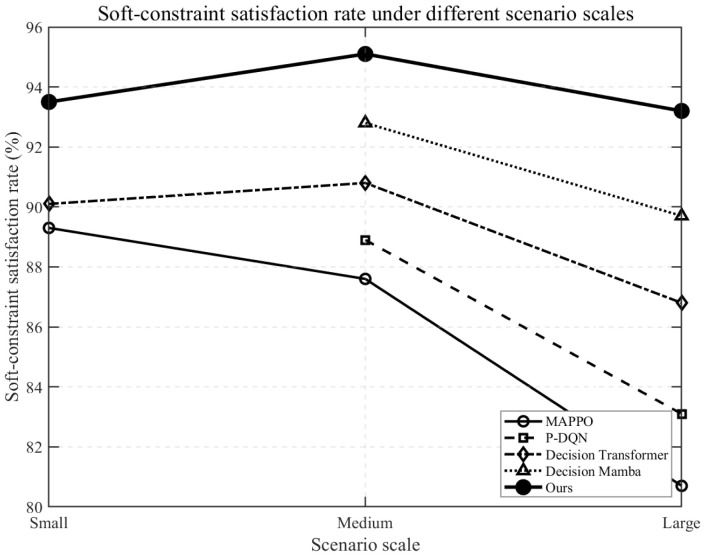
Comparison of soft-constraint satisfaction rates under different scenario scales.

The results which are shown in Figure 2, Figure 3 and Figure 4, show that the proposed framework maintains strong performance as the task scale and constraint complexity increase. This benefit comes from three aspects. First, the Mamba encoder captures long-term causal dependencies among residual power, task refresh intervals, interference evolution, and tracking uncertainty. Second, the hierarchical hybrid action mechanism decomposes radar scheduling into task prioritization, radar–task matching, and power allocation, reducing the difficulty of direct hybrid action learning. Third, action masking and feasible-domain projection suppress infeasible exploration and improve engineering executability.

### 4.5. Results on Public Scheduling Benchmarks

To evaluate generalization ability beyond the radar simulator, the proposed framework was further tested on public JSP/FJSP scheduling benchmarks. Radar nodes were mapped to machines, radar tasks were mapped to jobs or operations, and hard constraints were mapped to machine availability, operation compatibility, and time-window restrictions. The original makespan-oriented scheduling objective was converted into a normalized cost-effectiveness objective that jointly considers task utility, resource cost, and constraint penalties.

Table 9 shows that the proposed method consistently achieves the highest normalized cost-effectiveness score across different agent scales. Compared with Transformer-RL, our method improves the normalized cost-effectiveness score from 0.74 to 0.78 in the single-agent setting and from 0.70 to 0.75 in the four-agent setting. These results indicate that the proposed scheduler is not limited to the radar simulation environment but can also generalize to standard scheduling problems with structured resource-task matching.

### 4.6. Complexity and Real-Time Analysis

To further evaluate deployment efficiency, we compare the parameter size, FLOPs, and single-step inference latency of different neural scheduling methods under the same input sequence length K=200 and batch size 1.

As shown in Table 10, the proposed method achieves a favorable balance between model size and inference efficiency. Compared with Transformer-RL, our method uses only 25.4% of the parameters and 16.1% of the FLOPs, while reducing inference latency from 34.5 ms to 12.8 ms. This advantage mainly results from the linear-complexity Mamba encoder, which avoids the quadratic attention cost of Transformer-based sequence models. The hierarchical action generation module also avoids the parameter explosion caused by directly outputting high-dimensional hybrid actions.

### 4.7. Ablation Study

To analyze the contribution of each component, we conduct ablation experiments by removing or replacing the Mamba encoder, the remaining reachable cost-effectiveness return $G_{k}$, the hierarchical action generation module, the hard-constraint action mask, and the long-horizon input.

Table 11 verifies the necessity of the proposed components. Replacing the Mamba encoder with LSTM decreases the normalized cost-effectiveness score from 0.78 to 0.65, demonstrating the importance of long-range temporal modeling. Removing $G_{k}$ reduces the performance to 0.71, indicating that future-oriented return guidance helps prevent short-sighted scheduling. Without hierarchical action generation, the normalized cost-effectiveness score drops to 0.69, confirming the importance of matching the policy structure with the hybrid nature of radar scheduling. Removing the action mask also causes a clear performance decline, showing that hard constraints should be enforced during action generation rather than only penalized after execution. Finally, reducing the horizon from 200 slots to 50 slots decreases performance to 0.68, further validating the necessity of long-term scheduling information.

## 5. Conclusions

This paper proposed CS-Mamba, a long-horizon constraint-aware collaborative scheduling framework for homogeneous multiple phased-array radars. The scheduling problem was formulated as a finite-horizon decision process with structured hybrid actions, where radar–task matching and transmit-power allocation are jointly optimized under hard operational constraints and soft mission-level requirements.

The proposed method combines Mamba temporal encoding, structured radar–task matching, feasible power projection, and two-stage policy optimization. By explicitly distinguishing hard feasibility constraints from soft mission-level requirements and by linking transmit power to radar detection and tracking performance, the proposed framework improves both scheduling effectiveness and executability.

Experiments on MRSched-Bench demonstrate that CS-Mamba achieves higher normalized cost-effectiveness scores and lower end-to-end decision latency than representative optimization-based, reinforcement-learning, hybrid-action, and sequence-modeling baselines. Additional ablation studies verify the contributions of Mamba temporal encoding, structured matching, feasible power projection, and behavioral-cloning-based initialization followed by actor–critic fine-tuning.

This study is limited to homogeneous radar networks and simulation-based evaluation. Future work will extend the framework to heterogeneous radar networks, hardware-in-the-loop validation, and deployment on resource-constrained edge processors.

## Figures and Tables

**Table 1:** Comparison with related scheduling and decision-making methods.

Method Family	Long Horizon	Hybrid Action	Hard Feasibility	Radar Physical Model	Online Fine-Tuning
MILP/ILP	Partial	Yes	Yes	Simplified	No
CMDP/CPO	Yes	Limited	Penalty-based	No	Yes
P-DQN	Partial	Yes	Usually no	No	Yes
MAPPO/QMIX	Partial	Limited	Mask or penalty	Usually no	Yes
Decision Transformer	Yes	Limited	No	No	Limited
Decision Mamba	Yes	Limited	No	No	Limited
Classical radar allocation	Limited	Yes	Yes	Yes	No
CS-Mamba	Yes	Yes	Matching + projection	Yes	Yes

**Table 2:** State variables used in MRSched-Bench.

Component	Symbol	Dimension	Meaning
Residual power	$P_{r,k}^{rem}$	$N_{R}$	Remaining radar energy budget
Beam availability	$b_{r,k}$	$N_{R}$	Whether radar *r* can execute a task
Radar load	$l_{r,k}$	$N_{R}$	Historical utilization of radar nodes
Target position	$q_{t,k}$	$2N_{T}$ or $3N_{T}$	Target spatial location
Target velocity	$v_{t,k}$	$2N_{T}$ or $3N_{T}$	Target motion state
Tracking covariance trace	$\mathrm{tr}(P_{t,k})$	$N_{T}$	Tracking uncertainty
Task priority	$\omega_{t,k}$	$N_{T}$	Mission priority
Deadline	$d_{t,k}$	$N_{T}$	Remaining task deadline
Refresh interval	$\delta_{t,k}$	$N_{T}$	Slots since last execution
Jamming level	$JNR_{r,t,k}$	$N_{R}N_{T}$	Jammer-to-noise ratio
Clutter level	$CNR_{r,t,k}$	$N_{R}N_{T}$	Clutter-to-noise ratio
Geometric accessibility	$g_{r,t,k}$	$N_{R}N_{T}$	Beam visibility and coverage

**Table 3:** Dimensions and settings of the Mamba temporal encoder.

Variable or Setting	Dimension or Value
Input state $s_{k}$	$D_{s}$
Embedding vector $x_{k}$	$D_{emb}=256$
Hidden state $h_{k}$	$D_{h}$
Output representation $z_{k}$	$D_{z}$
Number of Mamba blocks	3
State-space dimension	16
Sequence length in Medium scenario	200

**Table 4:** Scenario configurations of MRSched-Bench.

Parameter	Small	Medium	Large
Number of radars $N_{R}$	2	4	8
Number of targets	4–6	8–12	16–24
Candidate task categories $N_{T}$	10	20	40
Scheduling horizon *K*	100	200	400
Slot length Δt	10 ms	10 ms	10 ms
JNR range	0–15 dB	0–20 dB	5–25 dB
CNR range	0–10 dB	0–15 dB	5–20 dB
Training scenarios	1000	2000	3000
Test scenarios	100	100	100
Random seeds	5	5	5

**Table 5:** Reward and evaluation coefficients.

Coefficient	Value	Meaning
$\lambda_{C}$	0.35	Resource-cost weight in CE
$\lambda_{\Phi }$	0.25	Penalty weight in CE
$\lambda_{p}$	0.10	Transmit-power cost coefficient
$\beta_{tr}$	0.50	Cooperative tracking penalty coefficient
$\beta_{per}$	0.50	Periodic refresh penalty coefficient
$a_{d}$	1.00	Detection-probability slope
$\gamma_{0}$	10 dB	Detection SNR threshold

**Table 6:** MAPPO hyperparameters.

Hyperparameter	Value
Actor hidden size	256, 256
Critic hidden size	512, 256, 128
Optimizer	Adam
Actor learning rate	$3\times 10^{-4}$
Critic learning rate	$1\times 10^{-3}$
Discount factor	0.95
GAE parameter	0.95
PPO clipping ratio	0.2
Entropy coefficient	0.01
Value loss coefficient	0.5
Batch size	4096
Mini-batch size	512
PPO epochs per update	10
Gradient clipping	0.5
Training steps	$2.0\times 10^{6}$
Random seeds	5

**Table 7:** Constraint and task-level performance in the Medium scenario.

Method	CE ↑	HVR ↓	SSR ↑	TCR ↑	PUR ↑	Tracking Error ↓
MAPPO	0.62±0.02	0.0%	82.6±2.4%	78.3±2.8%	71.5±3.1%	0.46±0.03
P-DQN	0.64±0.02	0.0%	84.1±2.2%	80.2±2.5%	73.8±2.9%	0.43±0.03
Decision Transformer	0.67±0.02	0.0%	86.7±2.0%	83.5±2.3%	75.6±2.6%	0.39±0.02
Decision Mamba	0.72±0.02	0.0%	90.8±1.7%	87.9±2.0%	78.4±2.3%	0.34±0.02
CS-Mamba	0.78±0.01	0.0%	95.3±1.2%	92.6±1.5%	82.1±1.8%	0.27±0.01

**Table 8:** Performance comparison on MRSched-Bench. CE denotes the normalized cost-effectiveness score, and latency is measured in ms per decision step.

Method	Small		Medium		Large		Missing Reason
	CE ↑	Latency ↓	CE ↑	Latency ↓	CE ↑	Latency ↓	
EDF	0.53±0.02	1.2±0.1	0.51±0.02	1.2±0.1	0.48±0.03	1.3±0.1	–
RM	0.55±0.03	1.3±0.1	0.53±0.03	1.3±0.1	0.49±0.03	1.4±0.1	–
GA	0.61±0.02	18.6±1.2	0.60±0.02	22.5±1.5	TO	TO	Timeout
Tabu Search	0.63±0.02	15.9±1.1	0.61±0.02	20.4±1.4	TO	TO	Timeout
MILP/ILP	0.70±0.01	134.8±6.5	TO	TO	TO	TO	Timeout
DQN	–	–	0.58±0.03	18.2±0.9	–	–	Discrete action only
PPO	–	–	0.63±0.02	20.1±1.0	–	–	–
MAPPO	0.65±0.02	14.5±0.8	0.62±0.02	24.5±1.2	0.54±0.03	31.6±1.8	–
P-DQN	–	–	0.64±0.02	21.6±1.1	0.57±0.02	28.9±1.5	–
Decision Transformer	0.66±0.02	21.3±1.0	0.67±0.02	28.4±1.5	0.61±0.02	39.7±2.0	–
Decision Mamba	–	–	0.72±0.02	16.5±0.8	0.66±0.02	20.8±1.1	–
CS-Mamba	0.71±0.01	12.4±0.5	0.78±0.01	12.8±0.6	0.73±0.02	15.7±0.9	–

**Table 9:** Cost-effectiveness comparison on public scheduling benchmarks with different agent scales.

Method	1-Agent	2-Agent	3-Agent	4-Agent
GA	0.621	0.625	0.625	0.625
PPO	0.682	0.667	0.653	0.644
A2C	0.703	0.682	0.673	0.663
SAC	0.711	0.691	0.684	0.678
TD3	0.703	0.682	0.672	0.667
Double DQN	0.695	0.673	0.664	0.658
Rainbow DQN	0.706	0.683	0.674	0.668
GNN-RL	0.736	0.718	0.702	0.696
Transformer-RL	0.744	0.727	0.712	0.702
**Ours**	**0.783**	**0.772**	**0.764**	**0.757**

**Table 10:** Comparison of model complexity and inference latency.

Method	Params (M)	FLOPs (G)	Latency (ms/Step)
PPO	2.8	0.62	30.1
MAPPO	4.5	1.05	34.5
GNN-RL	5.2	1.28	34.0
Transformer-RL	12.6	5.42	34.5
**Ours**	**3.2**	**0.87**	**12.8**

**Table 11:** Ablation study on long-term and constraint-aware components.

Variant	Description	Cost-Effectiveness ↑
Full CS-Mamba	Complete framework	**0.78**
w/o Mamba Encoder	Replace Mamba with LSTM	0.65
w/o $G_{k}$	Remove future return guidance	0.71
w/o Hierarchical Action	Use direct joint action output	0.69
w/o Action Mask	Remove hard-constraint masking	0.66
Short Horizon K=50	Reduce history from 200 to 50 slots	0.68

## Data Availability

The public JSP/FJSP benchmark datasets used for auxiliary evaluation are available from standard scheduling benchmark libraries. The high-fidelity private radar simulation data cannot be publicly released due to confidentiality restrictions. However, the authors will release a reduced public version of MRSched-Bench, including normalized radar parameters, scenario-generation scripts, configuration files, pseudo-random seeds, and evaluation scripts, to support reproducibility.
